# In Vitro Circadian Clock Gene Expression Assessments in Mesenchymal Stem Cells from Human Infants: A Pilot Study

**DOI:** 10.3390/nu16010052

**Published:** 2023-12-23

**Authors:** Melissa L. Erickson, Devin Dobias, Madeline Rose Keleher, Dana Dabelea, Bryan C. Bergman, Josiane L. Broussard, Kristen E. Boyle

**Affiliations:** 1 Translational Research Institute, AdventHealth, Orlando, FL 32804, USA; melissa.l.erickson@adventhealth.com; 2 Department of Pediatrics, University of Colorado Anschutz Medical Campus, Aurora, CO 80045, USA; devin.dobias@gmail.com (D.D.);; 3 The Lifecourse Epidemiology of Adiposity and Diabetes (LEAD) Center, Aurora, CO 80045, USA; dana.dabelea@cuanschutz.edu; 4 Division of Endocrinology, Metabolism and Diabetes, University of Colorado Anschutz Medical Campus, Aurora, CO 80045, USA; bryan.bergman@cuanschutz.edu (B.C.B.); jboussa@colostate.edu (J.L.B.); 5 Department of Health and Exercise Science, Colorado State University, Fort Collins, CO 80011, USA

**Keywords:** circadian, obesity, maternal, mesenchymal stem cells

## Abstract

Background: Exposure to intrauterine obesity can disrupt clock gene rhythmicity in animal models. The aim of this pilot study was to determine if maternal obesity alters rhythmic expression of core clock in mesenchymal stem cells (MSCs) from umbilical cords of human infants born to mothers with obesity (Ob-MSC) vs. normal weight (NW-MSC). Methods: We compared in vitro rhythmic expression patterns of core clock (*BMAL1*, *CLOCK*, *PER2*) and clock-output (*NR1D1*), components in undifferentiated Ob-MSCs (*n* = 3) vs. NW-MSCs (*n* = 3). MSCs were harvested every 2 h, following a dexamethasone shock, for 30 h. Adipogenesis or myogenesis was induced in vitro and markers of adipogenesis and fat storage were assessed, respectively. Results: We detected significant rhythmicity in expression patterns of *BMAL1*, *PER2*, and *NR1D1* at the group level in Ob- and NW-MSCs (*p* < 0.05). PER2 oscillatory amplitude was 3-fold higher in Ob-MSCs vs. NW-MSCs (*p* < 0.006). During adipogenesis, Ob-MSCs had higher PPAR*γ* protein content (*p* = 0.04) vs. NW-MSC. During myogenesis, Ob-MSCs had higher saturated triacylglycerols (*p* = 0.04) vs. NW-MSC. Conclusion: Rhythmic expressions of *BMAL1*, *PER2*, and *NR1D1* are detectable in undifferentiated MSCs. Higher *PER2* oscillatory amplitude was paralleled by higher markers of fat storage during differentiation in Ob-MSCs vs. NW-MSCs, and supports that the core clock and cellular metabolism may be linked in infant MSCs.

## 1. Introduction

Exposure to maternal obesity in utero is associated with obesity and metabolic disease later in life [1,2,3,4,5,6,7,8,9]. While the underlying biological mechanisms of these associations are not fully understood, it is posited that factors in the intrauterine environment have a direct effect on the metabolic function of fetal tissues. Mesenchymal stem cells (MSC) are progenitor cells for fetal mesodermal tissues that develop into adipose and skeletal muscle, which play a dominate role in whole-body metabolic health [10]. We have previously shown that MSCs isolated from umbilical cord tissue of infants born to mothers with obesity have lower total fatty acid oxidation during in vitro myogenesis, as well as greater fat content during adipogenesis and myogenesis, as compared to MSCs from infants born to mothers with normal weight [11]. Furthermore, fat content during differentiation corresponds to hypermethylation of genes involved in lipid metabolism (e.g., *PRKAG2*, *SDHC*) [11,12,13]. These findings support the notion that intrauterine exposures contribute to metabolic derangements as well as promote a fat storing phenotype in fetal MSCs, thereby increasing metabolic disease risk in offspring.

One potential mechanism by which the intrauterine exposures may impact metabolic function of fetal tissues is by modifying circadian molecular clocks. It is well accepted that maternal nutrition impacts offspring metabolism in important metabolic tissues such as skeletal muscle and adipose [14,15,16,17]. Virtually all cells in the body have an endogenous, self-sustaining molecular clock; the core of the clock mechanism is a network of transcriptional-translational feedback loops that generate rhythmic patterns of gene expression that oscillate in approximate 24 h cycles (e.g., a circadian rhythmic pattern of gene expression). The positive limb of the core clock consists of two promoters, basic helix-loop-helix ARNT like 1 (BMAL1) and clock circadian regulator (CLOCK), that heterodimerize and bind to E-box enhancer sequences to initiate the expression of the negative limb that consists of repressors period circadian regulator (PER)1/2 and cryptochrome circadian regulator (CRY)1/2. This primary loop is supported by a secondary loop consisting of nuclear receptor subfamily 1 group D member (NR1D)1/2 and retinoic acid-related orphan receptor (ROR*α*) [18]. Although the core clock feedback loop is a self-sustaining, it is also regulated by cellular energy status, which has consequences on systemic metabolic physiology [19,20]. For example, core clock disruption in central tissues leads to obesity [21] and *BMAL1* knockout in skeletal muscle leads to glucose intolerance [22]. In fact, Hansen et al. report the rhythmic oscillatory amplitude of *NR1D1* in human primary myotubes is related to the metabolic health of the donor [23]. These studies in cultured myotubes also show rhythmic expression of metabolic genes, such as Sirtuin (SIRT)1, a protein deacetylase regulating cellular energy metabolism and redox pathway proteins, which is linked to in vivo health outcomes and physical activity levels [23]. Further mechanistic work in mouse embryo fibroblasts has revealed that the core clock loop is regulated by *SIRT1*, providing the mechanistic link whereby the core clock is intertwined with cellular energy status [24]. The metabolic enzyme glycogen synthase kinase-β (gene name *GSK3B*) also directly interacts with the core clock feedback loop in neuronal tissue [25], further supporting mechanistic links between core clock activity and energy metabolism.

In vitro, acute palmitate exposure dampens the rhythmic oscillatory amplitude of core clock gene expression in primary myotubes derived from human volunteers [26], suggesting that nutrient exposures can directly influence circadian rhythms. In vivo, some evidence suggests that maternal nutrition during gestation can impact the core clock machinery in offspring peripheral tissues. For example, maternal dietary restriction in goats reduces *BMAL1* expression, a core component of the positive limb, in skeletal muscle of young offspring [27]. Likewise, high-fat feeding of pregnant rats reduced the area under the curve of core circadian gene expression (*CLOCK*, *NR1D1*, *CRY2*) in adult offspring liver [17]. Importantly, these animals also displayed disrupted metabolism and altered rhythmicity of *SIRT1* [17] and peroxisome proliferator-activated receptor-α (*PPARA*), a master regulator of lipid metabolism gene expressions [28], suggesting links between core clock gene disruption and metabolic gene dysregulation in the context of maternal nutrition. However, whether maternal obesity during gestation impacts the core clock or rhythms of metabolic genes in progenitor cells of human fetal tissue is unknown. 

Based on these previous observations showing rhythms in adult human myotubes, and disruption in animal models of maternal obesity of core clock and metabolic gene rhythmicity, the primary objective of this study was to assess in vitro rhythmic expression patterns of the core clock network in undifferentiated MSCs derived from human umbilical cords, which, to our knowledge, has not been conducted previously. We assessed the expression patterns of the core clock components *BMAL1* and *CLOCK* of the positive limb, *PER2* of the negative limb, as well as *NR1D1* of the secondary regulatory loop. We hypothesized that MSCs with intrauterine exposure to obesity, which have greater lipid stores and markers of adipogenesis compared with MSCs exposed to normal weight, would display dampened oscillatory patterns of molecular circadian clock gene expression. 

## 2. Materials and Methods

### 2.1. Mesenchymal Stem Cells

MSCs are from a subset of mother–infant dyads from the *Healthy Start Study*, in which maternal metabolic phenotyping data [6] was previously published. MSC outcomes including DNA methylation and lineage-specific metabolic phenotyping for lipid accumulation and fatty acid oxidation with adipogenesis and myogenesis were previously assessed [11]. From this initial cohort, we selected MSCs from three participants in the obesity group with previously defined hypermethylation of genes involved in lipid metabolism and MSCs from three participants in the normal weight group with hypomethylation of genes involved in lipid metabolism [13]. 

### 2.2. Participant Measures

Data collected in the Healthy Start Study included demographics, tobacco use, height, and weight. As described, women were characterized at mid-gestation (median of 17 weeks) and fasting blood samples were analyzed for glucose, insulin, triglycerides, and free fatty acids (FFA) at the University of Colorado Hospital Clinical and Translational Research Center Core Laboratory [6]. Inclusion criteria were 16 years of age or older, currently pregnant with a singleton carry (i.e., not pregnant with multiple fetuses), and ≥23 weeks of gestation. Exclusion criteria were prior diabetes, premature birth, and serious psychiatric illness. 

Infant birth weight was obtained through medical records. Body composition was assessed within 24–48 h after birth using whole body air plethysmography (PEA POD, COSMED, Inc., Concord, CA, USA). Mixed cord blood was collected at birth and assessed for glucose, insulin, triglycerides, FFA, and adiponectin [6].

### 2.3. Mesenchymal Stem Cell Culture 

**Figure 1:**
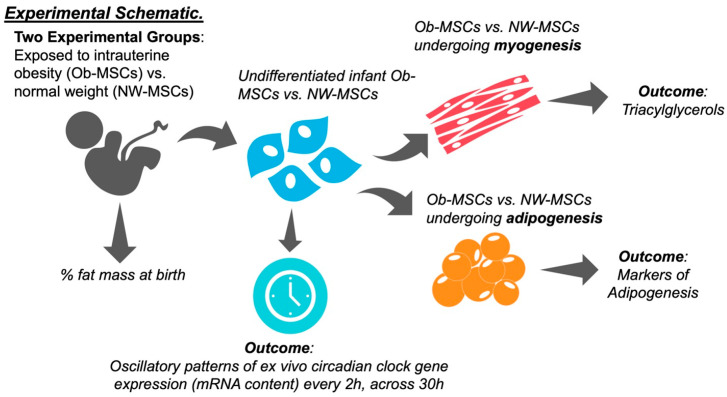
Experimental schematic. MSCs were isolated from umbilical cords of infants from mothers with normal weight or obesity. Gene expression was assessed in undifferentiated NW-MSCs and Ob-MSCs. Cell-based phenotyping was conducted in NW-MSCs and Ob-MSCs differentiating into myotubes and adipocytes.

The experimental schematic is presented in Figure 1. MSC culture and isolation procedures have been described previously [12]. In brief, plastic adherent progenitor cells were cultured from fresh umbilical cord explants in low-glucose Dulbecco’s Modified Eagle Medium (LG-DMEM, Corning Life Sciences, Corning, NY, USA) supplemented with MSC-qualified fetal bovine serum (FBS; Gibco, ThermoFisher Scientific, Waltham, MA, USA) and antibiotic/antimycotic. Cells were characterized as >98% for MSC markers CD74, CD105, and CD90 and negative for other cell types (CD34, CD45, CD19). Cells were cryopreserved and passaged and were within passages 5–7 for the experiments in this study.

### 2.4. Rhythmic Gene Expression Patterns in Mesenchymal Stem Cells

We assessed in vitro rhythmic expression patterns of metabolic genes under transcriptional control of the core clock including *GSK3B*, *PPARA*, and *SIRT1*. Undifferentiated MSCs were shocked for 1 h with (100 nM) dexamethasone (Sigma-Aldrich, St. Louis, MO, USA) followed by a 12 h rest period. Cells were then harvested every 2 h for a total of 30 h. Rhythmic expression patterns of components from the circadian clock network were assessed using quantitative PCR. Specifically, this included components from the core clock, *BMAL1*, well as *CLOCK*, and *PER2*, the secondary regulatory loop of the core clock, *NRID1*, and metabolic genes under transcriptional control of the circadian clock network, *SIRT1*, *PPARA*, and *GSK3B*, using quantitative PCR. 

### 2.5. RNA Isolation and qPCR 

Cells were harvested in Buffer RLT Plus (Qiagen, Germantown, MD, USA). Total RNA was isolated using RNeasy Plus mini kit (Qiagen, Germantown, MD, USA) and cDNA transcribed from 1 μg total RNA using iScript cDNA Synthesis kit (Bio-Rad Laboratories, Hercules, CA, USA). qPCR was performed using Taqman Gene Expression Assays with Taqman Fast Advanced Mastermix (Thermo Fisher, Waltham, MA, USA) for genes of interest with GUSB and PP1B as reference genes with a no-template control per gene. For MSCs differentiating into adipocytes, adiponectin mRNA expression was measured, with *GUSB* and *PP1B* as reference genes. Reactions were run in triplicate on a Viia 7 Real Time PCR System (Thermo Fisher, Waltham, MA, USA). Data were normalized using the geometric mean of the reference genes by ddCt method. Gene IDs and primer information are listed in Supplemental Table S1. 

### 2.6. Mesenchymal Stem Cell Differentiation 

MSCs underwent myogenesis or adipogenesis differentiation for 21 days as previously described [11,12]. In brief, myogenesis was induced using LG-DMEM supplemented with FBS, horse serum (Gibco, ThermoFisher, Waltham, MA, USA), dexamethasone, and hydrocortisone (Sigma-Aldrich, St. Louis, MO, USA). Adipogenesis was induced with LG-DMEM supplemented with FBS, dexamethasone, indomethacin, 3-isobutyl-1-methylxanthine, and human insulin (all, Sigma-Aldrich, St. Louis, MO, USA), treated alternately for 3 days each with LG-DMEM supplemented with FBS and insulin only. After 21 days, cells were collected for protein or fat content measures or exposed to experimental conditions for FAO assays, as previously described [11,12].

### 2.7. Triacylglycerol Content

We measured markers of adipogenesis and fat storage in Ob-MSCs and NW-MSCs undergoing differentiation into adipocytes and myotubes. Cell pellets were collected from 21 d myogenically differentiating cells and triacylglycerol (TAG) lipid species analyzed, as previously described [29]. Briefly, cells were lysed in PBS and then fortified with internal standards (ISs). Lipid extracted and analyzed by the Colorado Nutrition Obesity Research Center lipidomics core. Samples were run on an Sciex 2000 triple quadrupole mass spectrometer (Framingham, MA, USA). Concentration was determined by comparing ratios of unknowns to odd chain or deuterated internal standards and compared to standard curves run with standards of each lipid species.

### 2.8. Protein Content

Cells were harvested from 21 d adipogenic differentiating cells in lysis buffer (CelLyticTM MT, Sigma-Aldrich, St. Louis, MO, USA) supplemented with protease and phosphatase inhibitor cocktails (Sigma-Aldrich, St. Louis, MO, USA). Total protein was determined by BCA assay. Protein content of peroxisome proliferator-activated receptor (PPAR)*γ* with β-actin (Cell Signaling Technology, Danvers, MA, USA) as reference control was assessed by Simple Western size-based protein assay (WES, ProteinSimple, Santa Clara, CA, USA) following the manufacturer’s protocol. Results from WES were analyzed using ProteinSimple Compass software, v 6.1.0. Antibodies were optimized in-house for this system and antibody specifics and assay conditions are listed in Supplemental Table S2.

### 2.9. Statistical Analysis

Participant demographic data are presented as mean ± SD. Student’s unpaired, two-tailed *t*-tests were used to compare participant characteristics in NW vs. Ob groups. All cellular data are presented as mean ± SE unless otherwise noted. Circadian rhythmicity of gene expression was evaluated using CircWave v 1.4, which determines goodness of fit to a cosine wave. For gene targets in which circadian rhythmicity was significantly detected, circadian amplitude was subsequently determined. Student’s two-tailed *t*-tests were used to compare group differences in circadian oscillatory amplitude of gene expression between NW-MSCs vs. Ob-MSCs. Student’s two-tailed *t*-tests were also used to compare group differences in cell-based assays between NW-MSCs vs. Ob-MSCs. We conducted preliminary comparisons of expression patterns of core clock components and metabolic clock-output genes in MSCs derived from infants born to mothers with obesity (Ob-MSC) versus normal weight (NW-MSC) to represent diverse in utero exposures using Pearson correlation analyses to test for associations between outcomes of interest. Statistical analyses were conducted with GraphPad Prism (v 9.5.1).

## 3. Results

### 3.1. Participant Characteristics

Data are presented from 12 individual participants, resulting in 6 mother–infant dyads. Data on the methylation status of genes from MSCs from these participants were previously published as part of a larger dataset [11]. By design, mothers with obesity (Ob) had significantly higher BMIs than mothers with normal weight (NW; *p* = 0.002; Table 1). Additionally, maternal insulin was higher in the Ob as compared to the NW group (*p* = 0.049). Neonatal adiposity (g of fat and % fat mass) tended to be higher in infants born to mothers with obesity (*p* = 0.07 for both). 

### 3.2. Infant MSCs Have Detectible Rhythmic Expression Patterns in Components of the Circadian Clock Network

**Figure 2:**
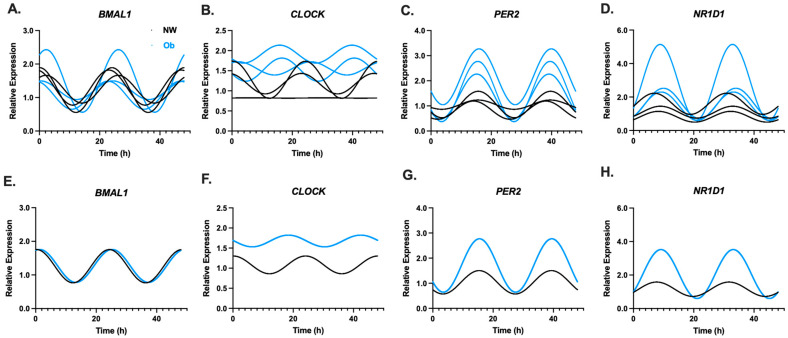
MSCs were harvested every 2 h for 30. Gene expression was measured, and the temporal patterns of expression were fit to a cosine wave and double plotted with *CircWave* for components of the circadian clock network, including components of the core clock (*BMAL1*, *CLOCK*, and *PER2*) and the secondary regulatory loop (*NR1D1)* in NW-MSCs and Ob-MSC at the individual (Top row; (**A**–**D**)) and group (Bottom row; (**E**–**H**)) level.

Rhythmic gene expression patterns of components from the core clock (*BMAL1*, *CLOCK*, *PER2*) and secondary regulatory loop (*NR1D1*) from NW-MSCs and Ob-MSCs are shown in Figure 2A–H. Significant circadian rhythms were detected in *BMAL1*, *PER2*, and *NR1D1* in both NW-MSCs and Ob-MSCs at the group level (Table 2). 

**Figure 3:**
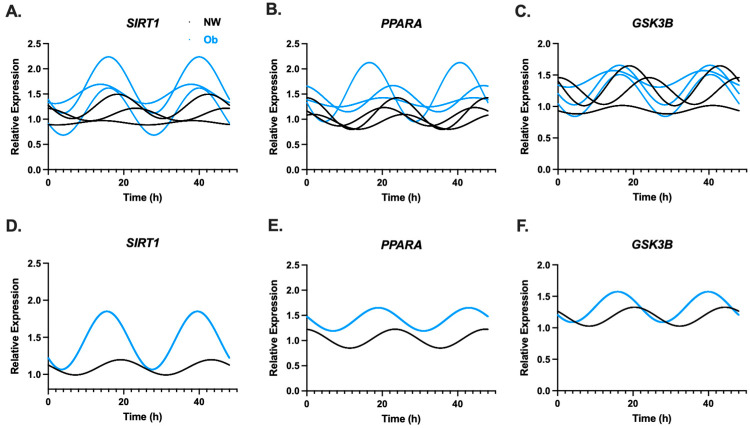
MSCs were harvested every 2 h for 30. Gene expression was measured, and the temporal patterns of expression were fit to a cosine wave and double plotted with *CircWave* for components for metabolic targets under transcriptional control of the circadian clock network (e.g., clock output) including *GSK3B*, *PPARA*, and *SIRT1* in NW-MSCs and Ob-MSC at the individual (Top row (**A**–**C**)) and group (Bottom row (**D**–**F**)) level.

Rhythmic gene expression patterns for metabolic gene under transcriptional control of the circadian clock network (*GSK3B*, *PPARA*, and *SIRT1*) from NW-MSCs and Ob-MSCs are shown in Figure 3A–F. Circadian rhythms were not detected in any metabolic genes examined (Table 2). 

### 3.3. PER2 Oscillatory Amplitude Is Higher in Ob-MSCs as Compared to NW-MSCs

**Figure 4:**
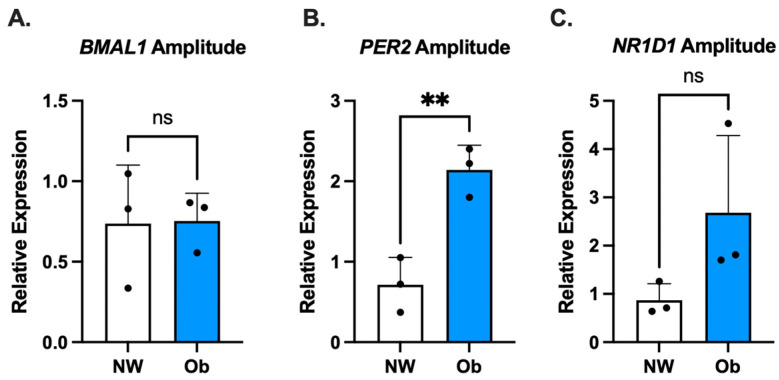
Oscillatory amplitude of circadian gene expression of *BMAL1* (**A**), *PER2* (**B**), and *NR1D1* (**C**) in NW-MSCs and Ob-MSCs. ns indicates not significant (*p* > 0.05) and ** indicates *p* < 0.01.

Circadian oscillatory amplitude was calculated for all genes whose expression displayed a significant circadian rhythm. There were no differences between groups in oscillatory amplitude for *BMAL1* or *NR1D1* (*p* = 0.95 and *p* = 0.13, respectively, Figure 4A,C). In contrast, the oscillatory amplitude of *PER2* was significantly higher in MSC-Ob as compared to MSC-NW (*p* = 0.006, Figure 4B). 

### 3.4. Ob-MSCs Have Greater Markers of Adipogenesis and Higher TAGs during Myogenesis

**Figure 5:**
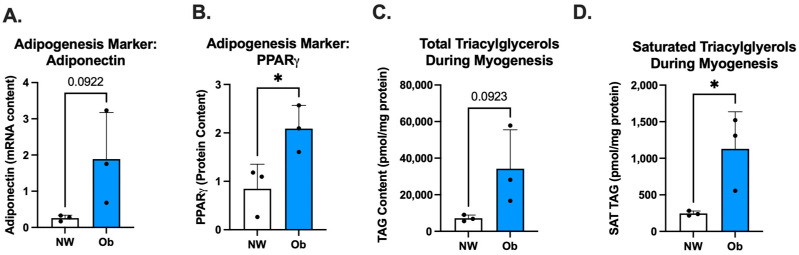
(**A**): Adiponectin mRNA content; and (**B**): PPAR*γ* protein content during adipogenesis; (**C**): Total triacylglycerols (TAGs) during myogenesis in NW-MSCs and Ob-MSCs; and (**D**): Saturated triacylglycerols (TAGs) during myogenesis in NW-MSCs and Ob-MSCs. * indicates *p* < 0.05.

During adipogenesis, Ob-MSCs had tended to have higher adiponectin mRNA content (*p* = 0.09; Figure 5A) and had higher protein levels of PPAR*γ* (*p* = 0.04, Figure 5B) compared to NW-MSCs. During myogenesis, Ob-MSCs had greater TAG content (*p* = 0.04; Figure 5C) and tended to have higher saturated TAG content (*p* = 0.09; Figure 5D) compared to NW-MSC. 

### 3.5. PER2 Amplitude May Be Linked to MSC Phenotype as Well as Maternal and Infant Traits

Associations between the oscillatory amplitude of *PER2* in undifferentiated MSCs and various outcomes are presented in Supplementary, including MSC markers of adipogenesis (Supplemental Figure S1), maternal insulin (Supplemental Figure S2), and neonatal adiposity (Supplemental Figure S3). *PER2* was selected for exploratory analyses based on the observed group differences in rhythmic amplitude between NW-MSCs and Ob-MSCs. *p*-values not reported due to limited sample size. 

## 4. Discussion

Circadian expression patterns of the molecular circadian clock network including core clock components *BMAL1* and *PER2*, as well as a component of the secondary regulatory loop, *NR1D1*, were present in MSCs derived from human umbilical cord tissue. To our knowledge, this is the first study to demonstrate circadian gene expression in human-derived primary stem cells prior to differentiation into tissue-specific lineages such as myotubes or adipocytes. One advantage of the MSC model is that it reflects infant phenotype in conjunction with in utero exposures, prior to environmental or lifestyle factors that occur after birth (e.g., diet, sleep/wake cycles) [10]. Thus, oscillatory rhythms of gene expression in MSCs may represent intrinsic circadian rhythms in infant tissues supporting the use of MSCs as an approach to investigate the impact of circadian regulation during human growth and development. 

The oscillatory patterns observed in components of the circadian clock network in MSCs were consistent with previous reports of other in vitro human derived cell-based assays. For example, data from experiments in primary human myotubes show that *BMAL1*, *PER2*, and *NR1D1* gene expression follows circadian rhythmicity in cells derived from groups of metabolically distinct participants [23]. Likewise, work in human progenitor cells derived from adipose tissue show robust oscillatory expression patterns of *BMAL1*, *PER2*, and *NR1D1* [30]. Similarly, in a rodent model of maternal obesity, adult offspring of obese dams show disruption to *CLOCK*, *NR1D1*, and *CRY2*, though this was mostly evident when offspring were weaned to a high fat diet [17]. Here, we found that *CLOCK* expression patterns were not rhythmic in MSCs, which is consistent with previous studies in human myotubes [23,26,31,32]. In addition, gene expression patterns of metabolic genes under transcriptional control of the core clock, including *SIRT1*, *PPARA*, and *GSK3B*, were not rhythmic in MSCs. This finding is also consistent with previous studies in human myotubes derived from sedentary lean, obese, and T2D phenotypes. Adult human myotubes and adipocytes originate from the fetal MSC lineage, and perhaps this explains the observed consistencies in cells from infants and adults. 

The circadian amplitude of *PER2* expression was higher in Ob-MSCs compared to NW-MSCs. This finding is somewhat inconsistent with previous reports of dampened oscillatory amplitude of *NR1D1* expression in primary myotubes from participants with T2D relative to myotubes from participants across the metabolic spectrum of obese, sedentary lean, and exercise trained lean [23]. Similarly, maternal obesity combined with a postnatal high fat diet reduces oscillatory amplitude of clock gene expression in mice offspring [17], contrary to the increased amplitude we observed here. The primary difference between the present study and previous reports of dampened oscillatory amplitude of clock gene expression in response to obesogenic exposures is that previous work was conducted in fully differentiated cells, whereas the present study used undifferentiated umbilical cord-derived MSCs. Our results showing increased PPAR*γ*, as well as increased storage of saturated TAG during myogenesis in Ob-MSCs [11,12,13], supports an elevated fat-storing phenotype during adipogenesis and myogenesis in parallel with higher *PER2* oscillatory amplitude, compared to NW-MSCs. Thus, perhaps higher *PER2* oscillatory amplitude is related more to the increased fat-storing ability of Ob-MSCs rather than maternal obesity per se. In support, increased *PER2* amplitude appears to correlate with markers of adipogenesis, including TAG content during myogenesis, as well as adiponectin and PPAR*γ* during adipogenesis. Given that insulin sensitivity is required for lipid storage, the MSC fat storing phenotype may also represent a more insulin sensitive state; thus, a higher *PER2* oscillatory amplitude in Ob-MSCs is not inconsistent with a dampened amplitude in myotubes derived from participants with impaired versus normal glucose tolerance [23]. However, we did not assess insulin sensitivity in MSCs in this study.

*PER2* is a core component in the repressor limb of the core clock feedback loop [18]. The higher *PER2* oscillatory amplitude we observed in undifferentiated Ob-MSCs vs. NW-MSCs, was accompanied by higher fat storage during experimental cellular differentiation. These observations support the relationship between core clock activity and cellular metabolism. Consistent with our cell-based assays, genetic knockout models of *PER2* show reduced triacylglycerol and non-esterified fatty acids, as well as a lean phenotype suggestive that the negative limb of core clock plays a role in whole-body fat storage [33]. In addition, *PER2* silencing leads to reduced lipid droplet accumulation and reduced cellular triglyceride content [34]. Future studies are warranted to examine the relationship between core clock, specifically robust circadian expression of *PER2*, and fat storage and adipose tissue insulin sensitivity. While we did not observe statistical links between *PER2* oscillatory amplitude and the other metabolic genes tested (*SIRT1*, *PPARA*, *GSK3B*), this may be potentially due to the small sample size of our preliminary study. 

Fetal exposures to excess metabolic substrates during maternal obesity are hypothesized to contribute to increased neonatal adiposity at birth [35], and our preliminary findings align with this hypothesis. For example, we observed that maternal insulin during late gestation may correlate with *PER2* amplitude in MSCs, suggesting that elevated insulin in utero may be one factor related to increased *PER2* circadian expression in fetal tissues. We also observed that *PER2* amplitude may correlate with neonatal adiposity at birth. This supports the notion that increased *PER2* circadian expression in fetal tissues is associated with fetal fat accretion, subsequently contributing to increased neonatal adiposity detectable at birth. These early findings set the stage for future hypothesis testing regarding the role of circadian pathways in the context of intergenerational obesity transmission. It is worth noting that in addition to maternal obesity-related exposures potentially impacting cellular rhythms, there may be other factors contributing to intrinsic infant MSC rhythm. These may include physical activity, dietary choices, sleep habits, or medications; however, this line of investigation is largely unexplored.

## 5. Conclusions

This study presents evidence for robust, cell autonomous, rhythmic oscillations of components of the molecular circadian clock network, which are detectable in vitro in undifferentiated MSCs derived from human infants. We also observed increased *PER2* oscillatory amplitude in Ob-MSCs compared to NW-MSCs. MSC phenotype reflects infant tissue; and therefore, this experimental approach serves as a useful tool for investigating circadian pathways that may contribute to metabolic disease risk in children exposed to obesity in utero. Future studies using larger sample sizes of MSCs from human infants are needed to better understand the role of the circadian clock network during fetal growth and development and relations to future obesity risk.

## Figures and Tables

**Table 1:** Characteristics of Mother–Infant Dyads.

	Normal Weight *n* = 3	Obese *n* = 3	*p*
Maternal Characteristics			
Age (yrs)	26.3 ± 6.4	33.7 ± 9.7	0.335
Pre-pregnancy BMI (kg/m^2^)	21.2 ± 1.7	32.3 ± 2.0	0.002 *
Race/ethnicity (self-reported), N			0.519
Hispanic	1	1	
White, non-Hispanic	1	2	
Black, non-Hispanic	0	0	
All others combined	1	0	
Fasting Plasma Glucose (mg/dL)	75.0 ± 3.6	74.3 ± 8.0	0.902
Fasting Plasma Insulin (mU/dL)	9.0 ± 2.0	13.7 ± 2.1	0.049 *
Infant Characteristics			
Sex (M/F)	2/1	2/1	--
Birthweight (kg)	3.2 ± 0.09	3.4 ± 0.3	0.244
Fat Free Mass (g)	2816.0 ± 154.3	2906.1 ± 260.7	0.632
Fat Mass (g)	191.7 ± 54.0	395.1 ± 136.0	0.074
Fat Mass (%)	6.4 ± 2.0	11.9 ± 3.4	0.074

Maternal fasting plasma assessments occurred during late gestation. MSC: Mesenchymal stem cells; Values are presented as average ± SD. * *p* ≤ 0.05.

**Table 2:** Circadian Rhythmicity of Gene Targets in Undifferentiated MSCs.

Gene Target	Normal Weight *n* = 3		Obese *n* = 3	
	R^2^	*p*	R^2^	*p*
*CLOCK*	0.117	0.136	0.019	0.716
*BMAL1*	0.347	0.001 *	0.222	0.021 *
*PER2*	0.367	0.001 *	0.505	0.000 *
*NR1D1*	0.229	0.012 *	0.444	0.000 *
*GSK3B*	0.059	0.345	0.086	0.241
*PPARA*	0.124	0.099	0.069	0.318
*SIRT1*	0.036	0.530	0.158	0.064

Circadian rhythmicity was assessed at the group level for NW-MSCs and Ob-MSCs. R^2^ represents goodness of fit to a cosine wave and *p*-value statistical significance of curve fit. Statistical tests were determined using *CircWave*. * *p* ≤ 0.05.

## Data Availability

The datasets available on request: The raw data supporting the conclusions of this article will be made available by the authors, without personal health or identifying information.

## Supplementary Materials

The following supporting information can be downloaded at: https://www.mdpi.com/article/10.3390/nu16010052/s1, Table S1: qPCR TaqMan Gene Expression Assay Information; Table S2: Antibodies for simple Western; Figure S1: Associations between PER2 amplitude and cell-based assays during adipogenesis; Figure S2: Associations between maternal insulin and PER2 amplitude; Figure S3: Associations between PER2 amplitude and neonatal adiposity.
