# Supplementary material for: In Vitro Circadian Clock Gene Expression Assessments in Mesenchymal Stem Cells from Human Infants: A Pilot Study
Source: Nutrients. 2023 Dec 23;16(1):52. doi: 10.3390/nu16010052 (PMC10780581; doi:10.3390/nu16010052)
Supplement: Supplementary file 1 [file nutrients-16-00052-s001.zip › nutrients-2750686-supplementary.pdf]

nutrients

MDPI

Supplementary Material

In Vitro Circadian Clock Gene Expression Assessments in Mesenchymal Stem Cells from Human Infants: A Pilot Study

Supplemental Table S1. qPCR TaqMan Gene Expression Assay Information

**Gene Target**	Taqman Assay
CLOCK	Hs00231857_m1
BMAL1	Hs00154147_m1
PER2	Hs01007553_m1
NR1D1	Hs00253876_m1
GSK3B	Hs01047719_m1
PPARA	Hs00947538_m1
SIRT1	Hs01009006_m1
ADIPOQ	Hs02564413_s1
GUSB	Hs00939627_m1
PP1B	Hs00168719_m1

Supplemental Table S2. Antibodies for Simple Western

**Target**	**Molecular Weight**	**Antibody**	**RRID**	**Antibody Dilution**	**Protein Concentration**
PPARγ	53, 57	Cell Signaling #2435	AB_2166051	1:25	0.8 mg/ml
β-actin	45	Cell Signaling #4970	AB_2223172	1:50	0.2 mg/ml

Supplemental Figure S1

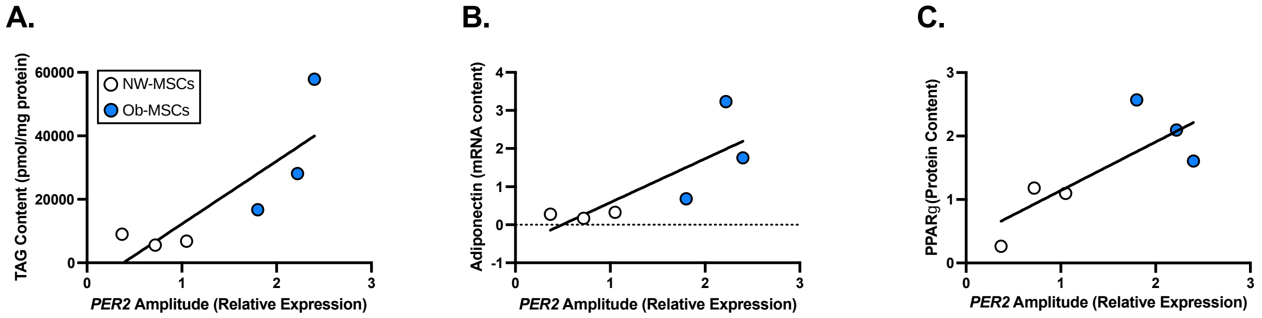

Figure S1: Associations between A: *PER2* amplitude and saturated TAGs during myogenesis; B: *PER2* amplitude and adiponectin during adipogenesis; and C: *PER2* amplitude and PPARγ during adipogenesis . NW-MSCs in open symbols and OB-MSCs in blue symbols.

Supplemental Figure S2

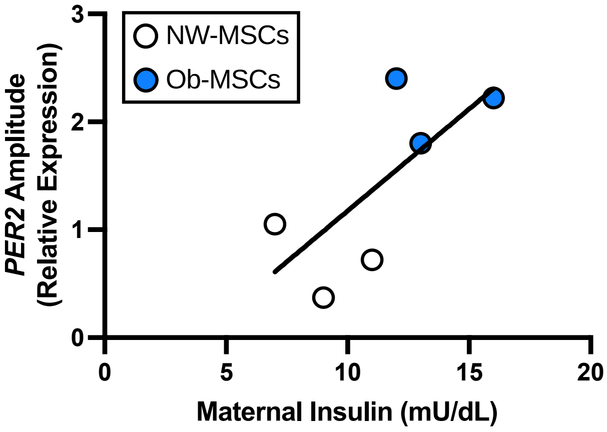

Supplemental Figure S2: Association between *PER2* amplitude and maternal insulin assessed during late gestation. NW-MSCs in open symbols and OB-MSCs in blue symbols.

Supplemental Figure S3

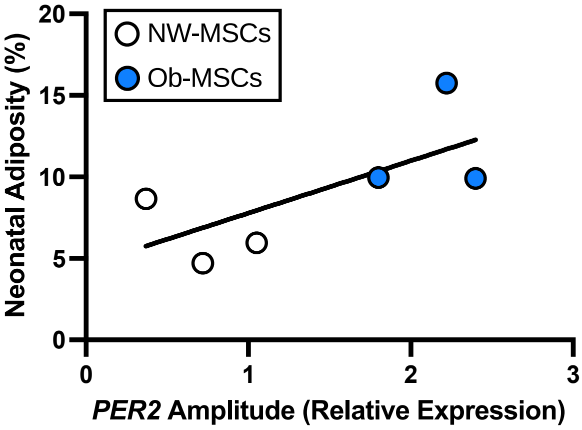

Supplemental Figure S3: Associations between *PER2* amplitude and neonatal adiposity. NW-MSCs in open symbols and OB-MSCs in blue symbols.
